# The clinical validity and utility of combinatorial pharmacogenomics: Enhancing patient outcomes

**DOI:** 10.1016/j.atg.2015.03.001

**Published:** 2015-03-24

**Authors:** Joachim Benitez, Michael R. Jablonski, Josiah D. Allen, Joel G. Winner

**Affiliations:** a Weill-Cornell Medical College, 1300 York Avenue, New York, NY 10065, United States; b Assurex Health, 6030 South Mason-Montgomery Road, Mason, OH 45040, United States; c Winner Psychiatry, PC., 2595 Canyon Boulevard, Boulder, CO 80302, United States

**Keywords:** Neurogenetics, Psychiatry, Combinatorial pharmacogenomics, Major depressive disorder

## Abstract

Prescribing safe and effective medications is a challenge in psychiatry. While clinical use of pharmacogenomic testing for individual genes has provided some clinical benefit, it has largely failed to show clinical utility. However, pharmacogenomic testing that integrates relevant genetic variation from multiple loci for each medication has shown clinical validity, utility and cost savings in multiple clinical trials. While some challenges remain, the evidence for the clinical utility of “combinatorial pharmacogenomics” is mounting. Expanding education of pharmacogenomic testing is vital to implementation efforts in psychiatric treatment settings with the overall goal of improving medication selection decisions.

## 1 Pharmacogenomics in psychiatry

Prescribing safe and effective medications is one of the greatest challenges in psychiatry. Large prospective clinical trials provide evidence for these challenges with increasing poor response rates and intolerance of treatment after a patient fails one medication, which are exacerbated with each subsequent medication failure (Warden et al., 2007). Pharmacogenomics (PGx) utilizes genetic information to predict responses to medications on a personalized level based on known gene–drug interactions for both pharmacokinetic (PK) and pharmacodynamic (PD) genes. Pharmacokinetic response to neuropsychiatric medications is largely driven by the cytochrome P450 system of enzymes expressed in the liver. Metabolism of medications by these enzymes affects the drug levels in the blood which will ultimately result in potential for efficacy and/or side effects. For example, an individual can be a poor metabolizer for a drug which will result in high drug blood levels and increase the potential for side effects. Pharmacodynamic genes predict response to a medication at the site of action. As an example, the serotonin transporter is encoded by SLC6A4 and has two main allelic variations, S and L. Patients with the S/S genotype have reduced rates of remission and response with selective serotonin reuptake inhibitor (SSRI) treatment (Porcelli et al., 2012).

Both pharmacokinetic and pharmacodynamic genetic differences contribute to the variability of response and potential for side effects for a patient. Select medications are exclusively metabolized by a single gene and will likely only be pharmacokinetically impacted by genetic variation at that single locus. For example, CYP2D6 copy number variation has been demonstrated to have a significant impact on plasma levels of nortriptyline, which is exclusively metabolized by CYP2D6 (Dalén et al., 1998). However, the majority of medications are metabolized by multiple enzymes, and can have considerable variability in receptor and transporter binding profiles. Given this complexity, it is unsurprising that single gene testing has shown limited clinical benefit for patients in ‘real world’ settings (Evaluation of Genomic Applications in Practice and Prevention (EGAPP) Working Group, 2007). However, synchronously evaluating the combination of PK–PK, PK–PD, and PD–PD gene interactions for a given individual has improved the clinical effectiveness of pharmacogenomic testing (Altar et al., 2015, Hall-Flavin et al., 2012, Hall-Flavin et al., 2013, Winner et al., 2013). For example, the SSRI citalopram is metabolized by 3 different enzymes (CYP2C19, CYP2D6, and CYP3A4) (Olesen and Linnet, 1999). Additionally, the serotonin transporter (SLC6A4) is the pharmacodynamic site of action for citalopram (Mrazek et al., 2009). Identifying the genotypic differences concomitantly for these four genes will lead to a combinatorial composite phenotype for citalopram and yield the most accurate clinically ‘actionable’ information. This combinatorial process allows the healthcare provider to assimilate complex layers of genetic information into information that supports medication decisions.

## 2 Clinical validity

Criteria for implementation of PGx information have been proposed (Hall-Flavin et al., 2013, Rundell et al., 2011), including ability to predict intolerable or non-efficacious treatments (clinical validity), simple integration into clinical practice (ease of use), and effective predictions to improve clinical outcome (clinical utility).

Clinical validity is the accuracy of a genetic test to predict a stated clinical outcome such as the likelihood of response to treatment. Three prospective clinical trials (two open-label trials and one placebo-controlled double-blind trial) have demonstrated the clinical validity of treatment guided by combinatorial pharmacogenomics in Major Depressive Disorder (Hall-Flavin et al., 2012, Hall-Flavin et al., 2013, Winner et al., 2013). Each of the three studies utilized GeneSight Psychotropic as the combinatorial PGx tool for physicians to help guide medication selection decisions. Based on an algorithm, the GeneSight report categorizes medications into three possible advisory categories: Use as Directed; Use with Caution; or Use with Increased Caution and with more Frequent Monitoring, which are color coded green, yellow, and red, respectively.

**Fig. 1:**
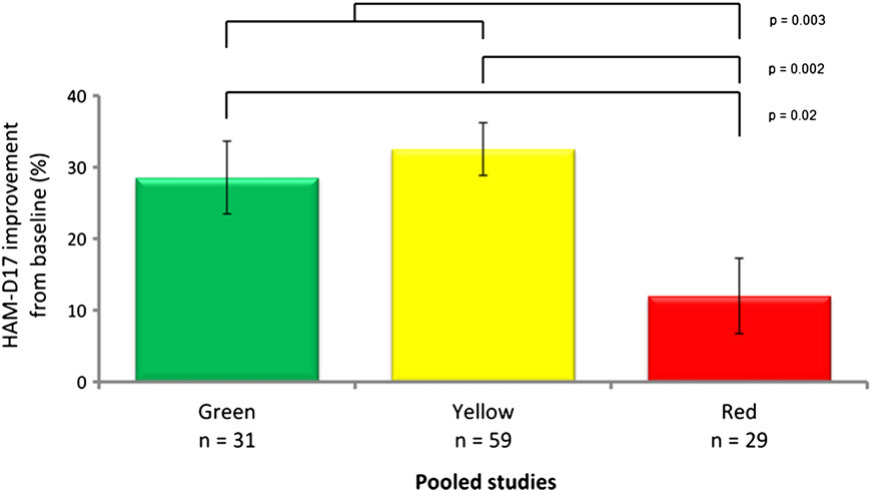
Clinical outcomes of blinded subjects treated without pharmacogenomic testing by GeneSight advisory category (pooled data from three trials).

The outcomes of the standard of care groups were analyzed to assess the clinical validity and predictive capability of the pharmacogenomic report. In the standard of care groups, the patients were blinded to their genetic information. However, after completion of the study, the PGx information was used to determine the clinical validity (predictive ability of response and intolerable treatment). In all three prospective clinical trials patients on red category medications, indicating a negative gene–drug interaction, experienced the worst outcomes based on the 17-item Hamilton Rating Scale for Depression (HAM-D17; Fig. 1). Moreover, in a pooled analysis of the three studies, combinatorial pharmacogenomics showed this ability to predict poor outcomes, whereas single genes were not predictive (Altar et al., 2015). The clinical validity of the GeneSight combinatorial PGx information is shown by its predictive capability to determine that patients who are placed on red category medications have increased likelihood of poorer outcomes.

## 3 Clinical utility

Clinical utility represents the improvement in outcomes in PGx tested individuals compared to standard of care. As with the clinical validity of PGx testing, three clinical trials have studied the clinical utility of combinatorial PGx information compared to standard of care. The first prospective, open-label trial identified a significant reduction in the GeneSight guided group compared to the standard of care group based on the HAM-D17 as well as the 16 item Clinician Rated Quick Inventory of Depressive Symptomatology (QIDS-C16). This was replicated by a much larger study, which resulted in a significantly improved response on the QIDS-C16 and HAM-D17, as well as the patient reported 9 item Patient Health Questionnaire (PHQ-9), in the GeneSight guided group compared to standard of care (Hall-Flavin et al., 2013). Finally, the smaller placebo-controlled, double-blind study trended towards similar clinical significance showing improvement in the GeneSight group compared to standard of care with double the likelihood of response (J. G. Winner et al., 2013).

## 4 Health economic outcomes

A number of trials have assessed the health resource utilization and potential cost savings for patients with the use of genetic reports. In a retrospective review, Chou et al. (2000) found that, among patients on CYP2D6-dependent medications, those with poor or ultrarapid CYP2D6 metabolism spent between $4000 and $6000 more in a one year period. Ruaño et al. (2013) retrospectively found that individuals with reduced CYP2D6 function averaged hospitalization length of stay that was two days longer than those with normal or increased CYP2D6 function. In a retrospective chart review using the GeneSight Psychotropic report, patients that were on medications predicted to yield the greatest gene–drug complications (i.e. “red category” medications) presented with significantly increased total health care visits, medical absence days, and disability claims compared to patients taking green or yellow category medications, resulting in nearly $5200 greater healthcare expenditures than those on genetically appropriate medications (J. Winner et al., 2013).

Most recently, larger scale analyses of health claims data have also been performed. One retrospective analysis of health claims data analyzed adherence rates and medication costs using a multi-genic (though not combinatorial) report (Fagerness et al., 2014). Patients provided with genetic testing (n = 227) were significantly more adherent to their medications compared to standard of care (n = 454); however, pharmacy costs increased in both the control and the genetically guided groups.

The largest economic study to date on combinatorial PGx testing (n = 2166 patients with genetic testing and n = 10,880 standard of care controls) analyzed total medication expenditures with the GeneSight test compared to standard of care in a prospective design (Allen et al., 2014a). Similar to the aforementioned retrospective study (Fagerness et al., 2014), adherence rates improved in the tested group. However, in this study using combinatorial PGx information, change in pharmacy costs in the GeneSight group were $1035.60 lower per patient per year compared to the propensity matched standard of care group. These costs improved even more for non-psychiatrists, patients whose physicians followed the genetic report, and patients with anxiety disorders (Allen et al., 2014b).

## 5 Conclusions

Pharmacogenomics is a quantitative tool to address the challenge of selecting appropriate psychiatric medications. However, gene-by-gene testing has shown limited clinical utility (Evaluation of Genomic Applications in Practice, 2007, Altar et al., 2015) in psychiatry. Combinatorial pharmacogenomics is able to identify individuals who are on medication regimens that have significant gene–drug interactions based on information which simultaneously integrates multiple genetic factors. The combinatorial approach combined with a streamlined platform to present this information has demonstrated clinical validity and utility (Hall-Flavin et al., 2012, Hall-Flavin et al., 2013, Winner et al., 2013), as well as cost-effectiveness (Allen et al., 2014a, Allen et al., 2014b, Winner et al., 2013). While some challenges still remain (Hamilton, 2015, Howland, 2014), the clinical trial data highlighted here provide evidence for the potential widespread benefit of pharmacogenomics in the psychiatric patient population.

As a value proposition, an intervention which offers the potential for improved outcomes combined with ‘real world’ cost savings is noteworthy. Recognizing the ongoing accumulation of clinical data in the psychiatric pharmacogenomic field and the enticing potential of a win–win scenario (lower costs, better outcomes) for its patient population, the Center for Medicare Services extensively evaluated the clinical validity, clinical utility, and economic data presented here. After this comprehensive process, the Center for Medicare Services released a specific coverage decision for the combinatorial GeneSight Psychotropic test (Center for Medicare and Medicaid Services, 2014), thus increasing the affordability and access to combinatorial testing for patients. Additionally, multiple private insurance companies and the U.S. Department of Veterans Affairs have made decisions to cover the GeneSight combinatorial test. Expanding education of pharmacogenomic testing is vital to implementation of these strategies in psychiatric treatment settings with the overall goal of improving medication selection decisions.
